# Chitosan-Functionalized Hydroxyapatite-Cerium Oxide Heterostructure: An Efficient Adsorbent for Dyes Removal and Antimicrobial Agent

**DOI:** 10.3390/nano12152713

**Published:** 2022-08-07

**Authors:** Aisha A. Alshahrani, Ali Q. Alorabi, M. Shamshi Hassan, Touseef Amna, Mohamed Azizi

**Affiliations:** 1 Department of Chemistry, College of Science, Al-Baha University, P.O. Box 1988, Al-Baha 65799, Saudi Arabia; 2 Department of Biology, College of Science, Al-Baha University, P.O. Box 1988, Al-Baha 65799, Saudi Arabia; 3 Department of Chemistry, Faculty of Science and Arts, Al-Baha University, Qilwah 65941, Saudi Arabia; 4 Lab. Desalination and Water Treatment Valorisation (LaDVEN), Water Research and Technologies Center (WRTC), BP 273, Soliman 8020, Tunisia

**Keywords:** adsorption, water remediation, CS-HAP-CeO_2_ heterostructure, antibacterial, Congo red

## Abstract

The current research intended to employ a facile and economical process, which is also ecofriendly to transform camel waste bones into novel heterostructure for cleansing of diverse waste waters. The bones of camel were utilized for preparation of hydroxyapatite by hydrothermal method. The prepared hydroxyapatite was applied to the synthesis of cerium oxide-hydroxyapatite coated with natural polymer chitosan (CS-HAP-CeO_2_) heterostructure. Being abundant natural polymer polysaccharide, chitosan possesses exceptional assets such as accessibility, economic price, hydrophilicity, biocompatibility as well as biodegradability, therefore style it as an outstanding adsorbent for removing colorant and other waste molecules form water. This heterostructure was characterized by various physicochemical processes such as XRD, SEM-EDX, TEM, and FT-IR. The CS-HAP-CeO_2_ was screened for adsorption of various industrially important dyes, viz., Brilliant blue (BB), Congo red (CR), Crystal violet (CV), Methylene blue (MB), Methyl orange (MO), and Rhodamine B (RB) which are collective pollutants of industrial waste waters. The CS-HAP-CeO_2_ demonstrated exceptional adsorption against CR dye. The adsorption/or removal efficiency ranges are BB (11.22%), CR (96%), CV (28.22%), MB (47.74%), MO (2.43%), and RB (58.89%) dyes. Moreover, this heterostructure showed excellent bacteriostatic potential for *E. coli*, that is liable for serious waterborne diseases. Interestingly, this work revealed that the incorporation of cerium oxide and chitosan into hydroxyapatite substantially strengthened antimicrobial and adsorption capabilities than those observed in virgin hydroxyapatite. Herein, we recycled the unwanted camel bones into a novel heterostructure, which assists to reduce water pollution, mainly caused by the dye industries.

## 1. Introduction

The ecosphere encounters pure water crisis even in the 21st century. Unpolluted water is essential for the ecosystem; however, inattention and maladministration of natural water assets had considerably endangered the availability of pure water. Annually, water contamination and allied ailments prerogative affect approximately 2.1 million lives. Predominantly, various biological pollutants are accountable for water-related infections. The incidence of Coliforms is considered as indicator of fecal contagion. The eruptions of water-related microbial infections and inorganic materials are the main causes of loss of precious lives [1]. Admitting, not severely damaging in minute amounts, but they are the main pollutants once concentrated in water. Similarly, organic materials and prescription drugs etc., enter through different sources into water bodies [2]. Besides being aesthetic, the colored dyes are cancer-causing, and block light from entering aquatic systems. Numerous dyes pose a health risk since they are poisonous to both plants and animals [3]. Congo red (CR) is a benzidine, which is based on anionic disazo dyes. It is a human carcinogen. This dye is dangerous for both humans and the environment [4].

The waste-water treatment methods are very expensive. Bearing in mind, the probable claim for unsoiled water in Saudi Arabia, there is an imperative need for expansion of inexpensive materials for sanitization of contaminated waters. Therefore, current investigation visualizes the synthesis of economical material from Albaha camel bones. *Camelus dromedarius*, encompasses as dominant livestock of Arabian Peninsula. Due to appreciable consumption of camel meat in Saudi Arabia, huge amount of waste camel bones is being generated. These waste camel bones lead to hefty disposal costs and are major threat to health, aesthetic pleasure, and the environment. Therefore, in this study camel waste bones collected from Albaha region were utilized for the first time for purification of wastewater. Nevertheless, the chemical synthesis of hydroxyapatite (HAP) is complicated and expensive. Therefore, a very simple and low-cost procedure for the extraction of natural bone precursor HAP from waste camel bones is applied. The camel bone waste is a good natural source to develop hybrid nanocomposites which possess remarkable antimicrobial activity; hence, can be used to treat the household wastewaters such as kitchen, toilets, laundry etc. A plethora of studies have utilized animal bones for adsorption of mercury ions [5], removal of divalent heavy metals from wastewater [6], etc. Moreover, several organic/inorganic HAP composites are being utilized for removal of different dyes from waste waters. For instance, plain HAP, and composites of HAP have been used to adsorb CR dye [7]. Similarly, HAP doped with magnesium, sodium alginate was applied to remove Acid Yellow 220 [8]. Similarly, HAP-Chitosan composite is used for the elimination of CR, and HAP with polyalcohol is used to remove CR, MB, and MO [9]. It is reported in the literature that HAP is a noteworthy material for the elimination of toxic constituents from waste waters. Consequently, this study reports the synthesis of HAP from Albaha camel bones which were utilized for purification of wastewater particularly Saudi Arabia wastewater. The interesting properties of HAP [10] eases with opportunity to use it in numerous applications [11].

Similarly, chitosan (CS) can also be cast-off as an adsorbent to eliminate dyes owing to the occurrence of amino and hydroxyl clusters (which assist as energetic spots). CS is an abundant natural alkaline polymer polysaccharide. The exclusive chattels, for instance easiness, cheapness, water compatibility, biocompatibility, as well as biodegradability make it as an outstanding adsorbent for eliminating dyes [12,13,14,15,16]. The admirable functional groups of CS (amino and hydroxyl groups) craft it as a fabulous adsorbent for interaction with diverse dyes. Moreover, it possesses intrinsic biomedical applications such as CS is incorporated in dressings to reduce bleeding and as an antibacterial agent. CS can also be used to help to deliver drugs through skin [17,18]. Very recently, chitosan-*Ulva* *lactuca* composites for eliminating of Cd(II) ions from aquatic media have been employed which displayed outstanding efficacy [19]. Likewise, at present, the progressive semiconductor photocatalysis skill has been documented as an auspicious methodology to resolve water pollution and energy problems owing to its low-cost, eco-friendly, environment reusability and high removal efficiency characters. Semiconductor metal oxide nanophotocatalysts, such as CeO_2_, ZnO, SnO_2_, Bi_2_O_3_, and TiO_2_ have been widely used to eliminate the toxic pollutants in wastewater under different light sources [20]. In current times, cerium oxide nanoparticles have attracted increased attention owing to their shielded 4f electrons which is accountable for their captivating assets [21]. In an investigation, the synthesized CeO_2_ nanopowder, displayed extraordinary adsorption competence for elimination of diverse dyes at specific pH [22]. In particular, recently the CeO_2_ composites such as La_2_O_3_–CeO_2_–Fe_3_O_4_ nanofibers [23] and Ce/ZnO/CNFs catalysts exhibit good stability and reusability, which would be an economical and environmental friendly photocatalysts for various practical applications and can simply be recycled [20]. However, antagonistic characteristics viz., meager solubility of CeO_2_ can be overawed by packing CeO_2_ with biopolymer (such as chitosan, dextran, cyclodextrin, glucose and folic acid etc.,), therefore intensifies their miscibility and biocompatibility. 

Consequently, with regard to exploring inexpensive material for purification of wastewater, this study recommends making usage of cheap and environment-friendly approaches to transform camel bone waste into novel composites such as CS-HAP-CeO_2_ heterostructure. The CS-HAP-CeO_2_ will then be applied as a chemical and biological agent for water purification. Moreover, solving water pollution crisis, our scientific contribution will also assist in plummeting the environmental pollution by recycling camel waste bones of Albaha province which might be a serious cause of pathogenic diseases in the very near future. 

## 2. Materials and Methods

### 2.1. Preparation of Hydroxyapatite (HAP)

The camel bones were assembled from abattoir of Albaha city of Saudi Arabia. Extensive cleaning of bones was done following our earlier procedures [24], primarily, these mandibles were swept away extensively using water, also with acetone to exclude fat and superfluous impurities. Later on, bones were put in an oven at 170 °C for 48 h. Furthermore, it was calcined in a furnace at an elevated temperature of 700 °C to get HAP.

### 2.2. Synthesis of Chitosan Coated Cerium Oxide-HAP Heterostructure (CS-HAP-CeO_2_)

Cerium sulfate (5.6 g, anhydrous, 97%, Alfa Aesar, India) was added to 80 mL of water in a beaker. Then s sodium hydroxide solution (2 M) was added dropwise to attain pH~11. HAP (2 g) powder was mixed in the same solution with continuous stirring. About 10 mL of chitosan solution (500 mg of chitosan was liquefied in acetic acid solution, 1% *v*/*v* in water) was dissolved to it. At that time the final solution was shifted to the hydrothermal reactor and heated at 180 °C for 15 h. The prepared CS-HAP-CeO_2_ heterostructures were filtered and rinsed quite a few times with water and then with ethanol and kept at 90 °C overnight in an oven. 

### 2.3. Characterization

The crystallinity of HAP and CS-HAP-CeO_2_ heterostructures were examined by X-ray diffraction (XRD, Rigaku (Tokyo, Japan) D/Max-2550, λ = 0.154 18 nm). The morphology of the samples was depicted by scanning electron microscopy equipped with energy-dispersive X-ray (SEM-EDX, JEOL, Tokyo, Japan). The presence of samples functional group was performed by Fourier transform infrared (FTIR, Thermo Scientific, Waltham, MA, USA) spectrometer. The microscopic images of samples were obtained by transmission electron microscopy (TEM, Hitachi, Tokyo, Japan). 

### 2.4. Adsorption Studies

The adsorption study was conducted by batch method. The impact of various factors such as adsorbent dosage, contact time, pH, dyes concentration, and temperature on the removal efficiency of CR by CS-HAP-CeO_2_ heterostructure in the range (0.0025–0.1 g), (5–1440 min), (3–11 pH), (50–500 mg/L), and (25–45 °C) respectively, was tested. For adsorptive dye removal, 0.02 g of CS-HAP-CeO_2_ heterostructure adsorbent solution was thoroughly mixed with 50 mL aqueous dye solution of 50 mg/L in 100 mL conical flask. Dye pH was changed by the addition of 0.1 M HCl or 0.1 M NaOH and then placed on a shaker at 100 rpm at ambient temperature, and the samples were tested at specific time intervals. After equilibrium period, the solution was filtered and the concentration of CR, RB, MB, CV, BB, and MO dyes were observed by UV–visible spectrophotometer at maximum absorption wavelengths (λ_max_) of 500, 603, 665, 590, 592, and 468 nm, respectively. The removal efficiency (%) and adsorption capacity (mg/g) were determined using the following Equations (1) and (2), respectively [25].

(1) $$\mathrm{Removal}\;\mathrm{efficiency}\;\left(\%\right)=\frac{\left(C_{o}-C_{e}\right)}{C_{o}}\times 100$$

(2) $$\mathrm{Adsorption}\;\mathrm{capacity}\;\left(q_{e}\right)=\left(C_{o}-C_{e}\right)\times \frac{V}{m}\;$$

where *C_o_* is the initial concentration of dye and *C_e_* is the concentration of MB equilibrium time), *V* is the volume of dye solutions, L, and *m* is the adsorbent dosage, g.

### 2.5. Antimicrobial Activity

The antibacterial activity of pristine HAP and novel CS-HAP-CeO_2_ heterostructure was verified in liquid broth [26]. The *E. coli* culture was maintained (10^6^ CFU) with known amount of pristine HAP and CS-HAP-CeO_2_ heterostructure (0, 50, 100, and 200 µg/mL) to regulate the minimum inhibitory concentration (MIC). The expansion kinetics was supervised at every 4 h by reading the OD in a spectrophotometer. A persistent incubation temperature of 37 °C and rpm of 150 were sustained in a rotary shaker. Growth inhibition in presence of HAP and HAP-Ce coated with natural polymer CS was upheld to 16 h and the shift in absorbance was considered at 600 nm by ultra-violet [27] spectrophotometer. 

## 3. Results

### 3.1. Sythesis and Characterization

The formation of CS-HAP-CeO_2_ heterostructure can be hypothesized as follows: the bone does have 65–70% HAP and 30–35% organic compounds (on dry weight basis). In our work, we extracted HAP by simple thermal treatment of natural bone. Cerium oxide was prepared using cerium sulfate precursors with HAP in solution. The use of sodium hydroxide resulted in a white precipitate, Ce(OH)_3_, from cerium sulfate solution. Oxidation of Ce^3+^ to Ce^4+^ in solution occurs at high pH and forms Ce(OH)_4_ [28]. Then it is converted to cerium oxide at high temperature. Furthermore, chitosan as a linear polysaccharide consists of (1,4)-linked 2-amino-deoxy-b-d-glucan with two free hydroxyl and one primary amino group. The positive and negative charge of amino and hydroxyl groups could make it available to attached with the surface of HAP and cerium oxide, thus resulting in the formation of novel CS-HAP-CeO_2_ heterostructure [28]. 

**Figure 1:**
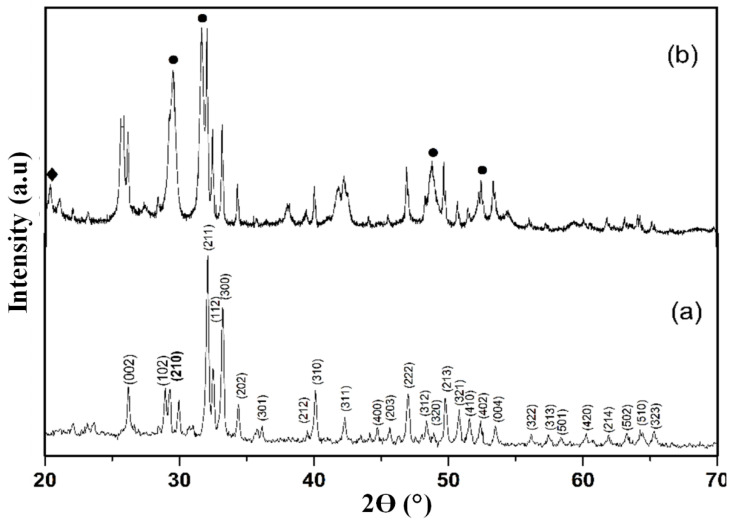
The XRD spectra of (**a**) pure HAP and (**b**) CS-HAP-CeO_2_ heterostructure. The symbol (●) indicates cerium oxide peaks whereas, (◆) represents chitosan peak.

Figure 1 displays the XRD spectra of untainted HAP and CS-HAP-CeO_2_ heterostructure. All diffraction peaks of pure HAP correspond to single phase hexagonal structure of [Ca_10_ (PO_4_)_6_(OH)_2_] (JCPDS no. 09-0432). There was no contamination peaks present in the spectrum, it confirms the formation of pure HAP [29] (Figure 1a). The XRD patterns of CS-HAP-CeO_2_ heterostructure exhibited all characteristic diffraction peaks of HAP, in addition, it also shows the diffraction peaks corresponding to the crystalline plane (111), (200), (220), and (311) which confirms the cubic fluorite assembly of CeO_2_ (JCPDS no. 81-0792) [30]. In the same spectrum, a weak peak at around 2θ of 20° corresponds to pure chitosan [31] (Figure 1b). 

**Figure 2:**
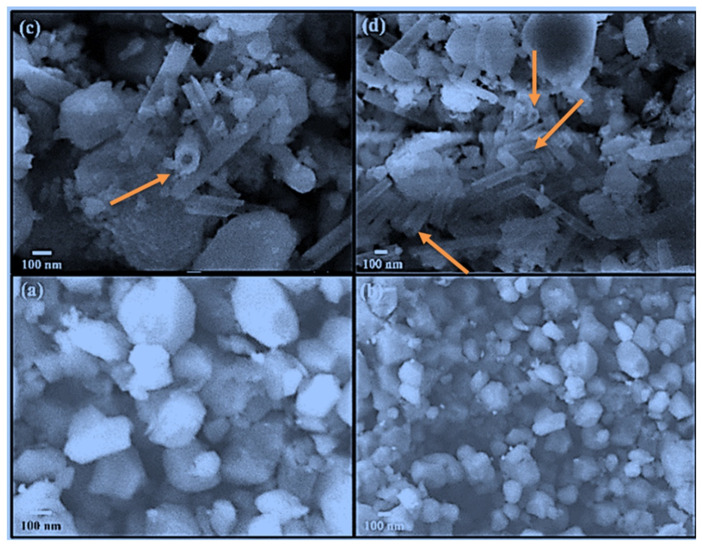
The SEM images of (**a**,**b**) pure HAP and (**c**,**d**) CS-HAP-CeO_2_ heterostructure. The arrows represent the CeO_2_ nanotubes embedded in HAP nanomatrix.

Figure 2 presents the surface structure and morphology of pure HAP and CS-HAP-CeO_2_ heterostructure. Figure 2 illustrates the micro-structural study of HAP particles, which shows somewhat spherical in shape, displaying small and large particles with maximum size of around 1 µm (Figure 2a,b). Whereas, in the heterostructure, the HAP particles together with nanotube-like structure having diameter of around 100 nm with varying lengths (Figure 2c,d) were visible.

**Figure 3:**
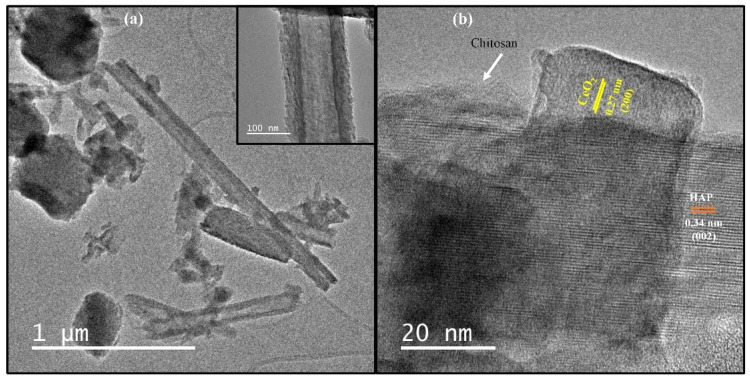
The (**a**) TEM (inset shows single nanotube) and (**b**) HR-TEM images of CS-HAP-CeO_2_ heterostructure.

The close inspection of the TEM image confirmed the presence of CeO_2_ nanotubes having diameter of around 100 nm over the surface of HAP particles, which is in good agreement with SEM results (Figure 3a). The high-resolution image clearly shows different crystal structures on the heterostructure surface, the fringes of d = 0.34 nm and d = 0.27 nm, observed in Figure 3b matched well to those of (002) and (200) crystallographic planes of HAP and CeO_2_ particles, respectively. A thin amorphous uniform coating layer on the crystal surface can also be seen confirming the chitosan presence in the heterostructure (Figure 3b). This confirms that CS-HAP-CeO_2_ heterostructure was successfully prepared.

**Figure 4:**
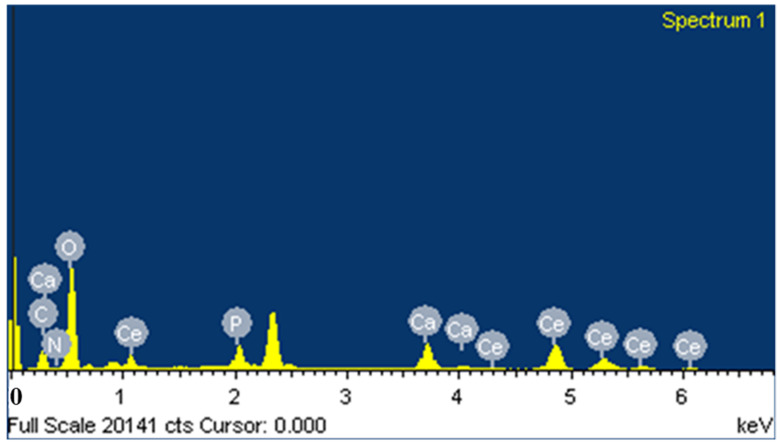
The EDX spectrum of CS-HAP-CeO_2_ heterostructure.

The EDX spectrum of CS-HAP-CeO_2_ showed the presence of all the expected elements in heterostructure. The EDX analysis confirms the presence of calcium (Ca), phosphorus (P), cerium (Ce), and oxygen (O) (Figure 4). 

### 3.2. Adsorption Studies

#### 3.2.1. Adsorbate Selectivity

**Figure 5:**
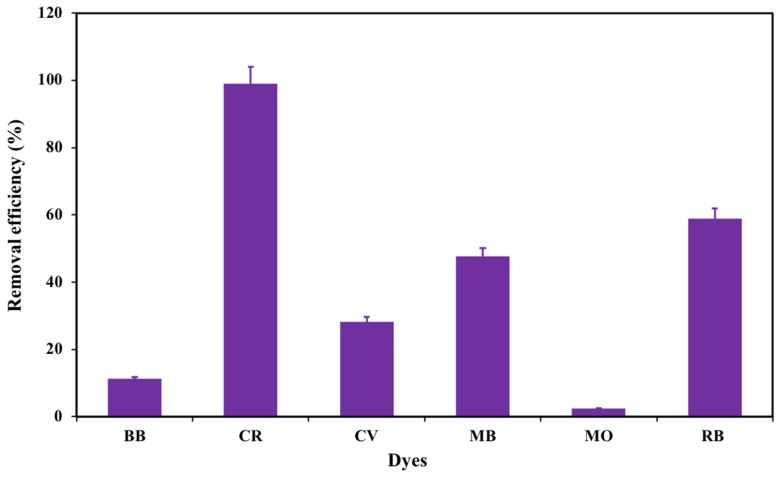
Selectivity studies of CS-HAP-CeO_2_ heterostructure toward different dyes.

The obtained adsorbent was tested for removal of various anionic dyes such as CR, RB, BB, and MO and cationic dyes such as CV and MB in aqueous medium at the parameters (dye = 20 mg/L, agitation = 100 rpm, dose = 0.02 g; and 25 °C) as shown in Figure 5. The experimental observations revealed that removal efficiency of the different dyes were BB (11.22%), CR (96%), CV (28.22%), MB (47.74%), MO (2.43%), and RB (58.89%). This indicates that the removal efficiency of CR dye was higher than the other tested dyes and order of affinity built on the amount of dye uptake was found to be: CR > RB > MB > CV > Bb > MO dye. This is owing to the fact that different dyes will undergo different physical and electrostatic forces, thus the difference in removal efficiency of dyes is due to their structure, molecular size, and functional groups which react with the functional groups of adsorbent surfaces with different physical and electrostatic forces [32,33]. Consequently, CR molecules occupy larger area over the CS-HAP-CeO_2_ heterostructure surface.

#### 3.2.2. Effect of pH 

**Figure 6:**
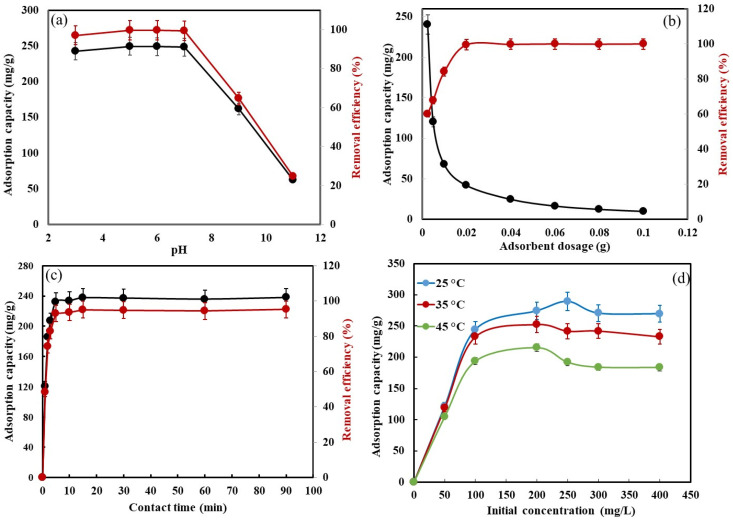
Effects of pH (**a**), adsorbent dosage (g) (**b**), contact time (min) (**c**), and initial concentrations (mg/L) (**d**) on the adsorption of CR dye onto CS-HAP-CeO_2_ heterostructure.

The initial pH solution shows an important role in the adsorption process owing to its impact on both, the active sites of ionization process of the CR molecule and the CS-HAP-CeO_2_ heterostructure in the solution. Therefore, the effect of pH solution on the adsorption process at (CR = 100 mg/L, agitation = 100 rpm, dose = 0.02 g; and 25 °C) was tested at the varying initial pH (3–11) as presented in Figure 6a. The CR is a dipolar molecule and it represents a cationic form at acidic pH and an anionic form at basic pH [34]. Moreover, the normal red color of CR at pH around 7 changes to dark blue in a highly acidic medium and to red in basic medium ~pH (11–12), this color in an alkaline medium is slightly different from the original red at pH = 7 [35]. The results reveal that the removal efficiency and adsorption capacity increased from (242.84 mg/g and 97.13%) to (248.40 mg/g and 99.63%) with the increase in pH from 3 to 7, respectively, and then reduced. The dissociation constant (pKa) of CR is 4.0 [36]. According to this value when the pH < (pKa = 4), the surfaces of both CS-HAP-CeO_2_ heterostructure and CR dye are positively charged, showing an electrostatic repulsion between them. On the other hand, when the pH > (pKa = 4), the CR dissociates into polar groups (SO_3_^−^) along with the positive charge of –N=N^+^– groups. The SO_3_^−^ group over the CR surface adsorbs on positively charged CS-HAP-CeO_2_ adsorbent by electrostatic attraction along with H-bonding. As pH increases to 7, the removal efficiency of CR was increased to 99.84% owing to the number of positive charges in CS-HAP-CeO_2_ heterostructure surface, decreasing which led to creation of electrostatic attraction between the –N=N^+^– group of CR molecules and CS-HAP-CeO_2_ heterostructure surface. At an alkaline medium, at high pH, the existence of excess ^−^OH ions compete with the CR anions (sulfonate groups) for the adsorption sites on the CS-HAP-CeO_2_ heterostructure surface, leading to a decrease in the removal efficiency of CR dye [37,38]. Therefore, at pH = 7, probably, electrostatic attraction and H-bonding become dominant in the adsorption mechanism. Thus, pH 7 was chosen as the optimum pH value in further experiments. The reported effect of pH on adsorption of CR dye by MgO composite [39] is in agreement with our study. Furthermore, a recent review report based on the applications of artificial intelligence has captured incredible attention owing to solve future water-related problems [40]. Moreover, the cellulose nanofibril/rectorite composite sponges has displaced an efficient dye adsorption and selective separation in recent investigation [41].

#### 3.2.3. Effect of Adsorbent Dose

The influence of CS-HAP-CeO_2_ heterostructure amount on CR dye adsorption at parameters conditions (CR = 20 mg/L, pH = 7; and 25 °C) in the range (0.0025–0.1 g) was studied as mentioned in Figure 6b. It was noticed that the removal efficiency of CR increases from 60.16% to 99.43% with the increase in amount of CS-HAP-CeO_2_ heterostructure adsorbent from 0.0025 to 0.02, and then became constant as the amount of CS-HAP-CeO_2_ heterostructure adsorbent increased to 0.1 g. An increase in the removal efficiency with the increase in amount of CS-HAP-CeO_2_ heterostructure adsorbent can be ascribed to the increasing accessibility of more adsorption sites [42]. On the other hand, the adsorption capacity was reduced from 240 mg/g to 20 mg/g with an increase in an adsorbent amount from 0.0025 to 0.1 g. This might be due to the aggregation events at high dosage [43]. Similar results were reported to the effect of adsorbent dose on adsorption of CR by *Teucrium polium* L. [44] and polypyrrole-modified red mud [45]. Therefore, 0.02 g was chosen as the optimal adsorbent dose under the examined conditions.

#### 3.2.4. Effect of Contact Time

Figure 6c shows the effect of contact time on the removal of CR dye at an initial concentration of 100 mg/L onto CS-HAP-CeO_2_ adsorbent under various conditions (dose–0.02 g; pH–7; T–25 °C). The present outcome exhibited that the adsorption capacity of the CS-HAP-CeO_2_ heterostructure and removal efficiency of CR dye increased rapidly from (120.91 to 233.65 mg/g) and (48.36% to 96%) at 100 mg/L with the increase in time from 1 to 15 min at the initial concentration of CR dye, respectively. As a result, the ideal time for equilibrium for future work was evaluated to be 15 min. The CR adsorption on graphene–chitosan composite hydrogel has shown a comparable contact time effect [46].

#### 3.2.5. Effect Temperatures

The effect of temperature on CR adsorption of dye on the CS-HAP-CeO_2_ adsorbent was investigated at different temperatures (25, 35, and 45 °C) and at different concentrations (50–400 mg/L). Figure 6d shows the initial CR dye concentration effect on the adsorption process at various concentrations (50 to 400 mg/L) and temperatures (25, 35, and 45 °C). The conditions are as follows: (dose–0.02 g; pH–7). When the CR dye concentration increased from 50 to 250 mg/L at 25 °C, the adsorption capacity of the CS-HAP-CeO_2_ adsorbent increased from 121.87 to 289.21 mg/g. The increase in the driving force for mass transfer from solution to the CS-HAP-CeO_2_ heterostructure adsorbent surface results in prominent increase in adsorption capacity at higher initial concentrations [47,48,49]. It was perceived that when the temperature rises, the adsorption capacity decreased marginally, indicating that the CR dye adsorption was an exothermic process. At higher temperatures, the binding forces on the surface with the CS-HAP-CeO_2_ heterostructure adsorbent were destroyed [50]. These results were consistent to that of CR adsorption on a fly ash/CeO_2_ composite adsorbent [51], malachite green and CR were removed from aqueous solution using magnetic HAP nanopowder [52], CR adsorption on graphene oxide/chitosan fibers [53], as well as CR adsorption on calcium HAP nanoparticles [54].

### 3.3. Adsorption Kinetics

**Figure 7:**
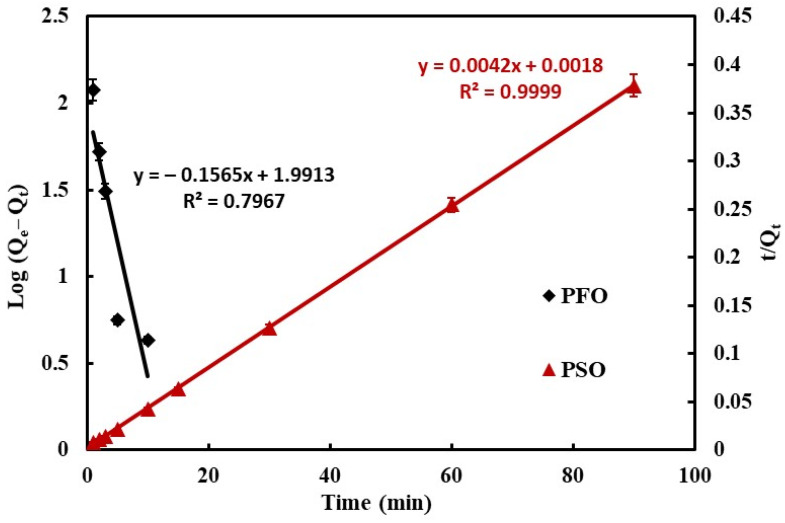
Plots of pseudo-first-order and pseudo-second-order kinetic model for adsorption of CR onto CS-HAP-CeO_2_ heterostructure adsorbents.

To determine the CR adsorption mechanism onto CS-HAP-CeO_2_ heterostructure, two most common adsorption kinetics namely, pseudo-first-order equation (PFO) (Equation (3)) and pseudo-second-order rate equation (PSO) (Equation (4)) were applied. The equations can be expressed as follows [55]:

(3) $$\log(q_{e}-q_{t})=\log(q_{e})-\frac{k_{1}}{2.303}t$$

(4) $$\frac{t}{q_{t}}=\frac{1}{k2q_{e}^{2}}+\frac{1}{q_{e}}t$$

where *k*_1_ (1/min) and *k*_2_ (g/(mg·min)) represent the PFO and PSO constant, respectively; *q_e_* and *q_t_* (mg/g) represent the adsorption capacity of the CS-HAP-CeO_2_ at equilibrium and at various times, respectively. The linear curves plotted of log (*q_e_* − *q_t_*) and (*t*/*q_t_*) versus *t* are displayed in Figure 7, and the kinetic parameters are listed in Table 1.

The results show the experimental data fitted very well with PSO kinetic model, because the R^2^ value for the PFO (R^2^ = 0.9999) is higher than the R^2^ value for the PFO (R^2^ = 0.7967). Moreover, the adsorption capacity (*q_e,cal_* = 238.09 mg/g) value derived from PSO is close to that obtained experimentally (*q_e,exp_* = 237.95 mg/g). Hence, the results show that the adsorption of CR dye onto CS-HAP-CeO_2_ heterostructure is chemical adsorption via electrostatic attraction and H-bonding. The same result was observed for adsorption of CR by EG@MnFe_2_O_4_ and MnFe_2_O_4_ [36].

### 3.4. Adsorption Isotherm

To determine the mechanism of the adsorption process and evaluate the quantity of CR dye adsorption onto the CS-HAP-CeO_2_ heterostructure, four linear isotherm models namely, Langmuir Equation (5), Freundlich Equation (6), Temkin (Equations (7) and (8)), and Dubinin–Radushkevich (Equations (9)–(11)) were used. The equations can be expressed as follows [36]:

(5) $$\frac{1}{q_{e}}=\frac{1}{q_{m}}+\frac{1}{q_{m}K_{L}C_{e}}$$

(6) $$\log q_{e}=\log K_{f}+\frac{1}{n}\log C_{e}$$

(7) $$q_{e}=B\ln A_{T}+B\ln C_{e}$$

(8) $$B=\frac{RT}{b_{T}}$$

(9) $$\ln q_{e}=\ln\left(q_{s}\right)-\left(K_{ad}\varepsilon^{2}\right)$$

(10) $$\;\;\;\;\;\;\;\;\;\;\;\;\;\varepsilon =RT\ln\left(1+\frac{1}{C_{e}}\;\right)$$

(11) $$E=\frac{1}{\sqrt{2\times K_{D}}}$$

where *C_e_* (mg/L) represents the equilibrium concentration of CR dye (mg/L), *q_m_* is the maximum monolayer adsorption capacity (mg/g), *K_ad_* (mol^2^/kJ^2^), *K_f_* (mg/g), and *K_L_* (L/mg), represent the Dubinin–Radushkevich, Freundlich, and Langmuir isotherm constant, respectively; *n* is the adsorption intensity; *A_T_* (L/g) and *B* (J/mol) are the binding constant and the constant related to heat of sorption. ε is the Polanyi potential and *E* (kJ/mol) is the average free energy.

**Figure 8:**
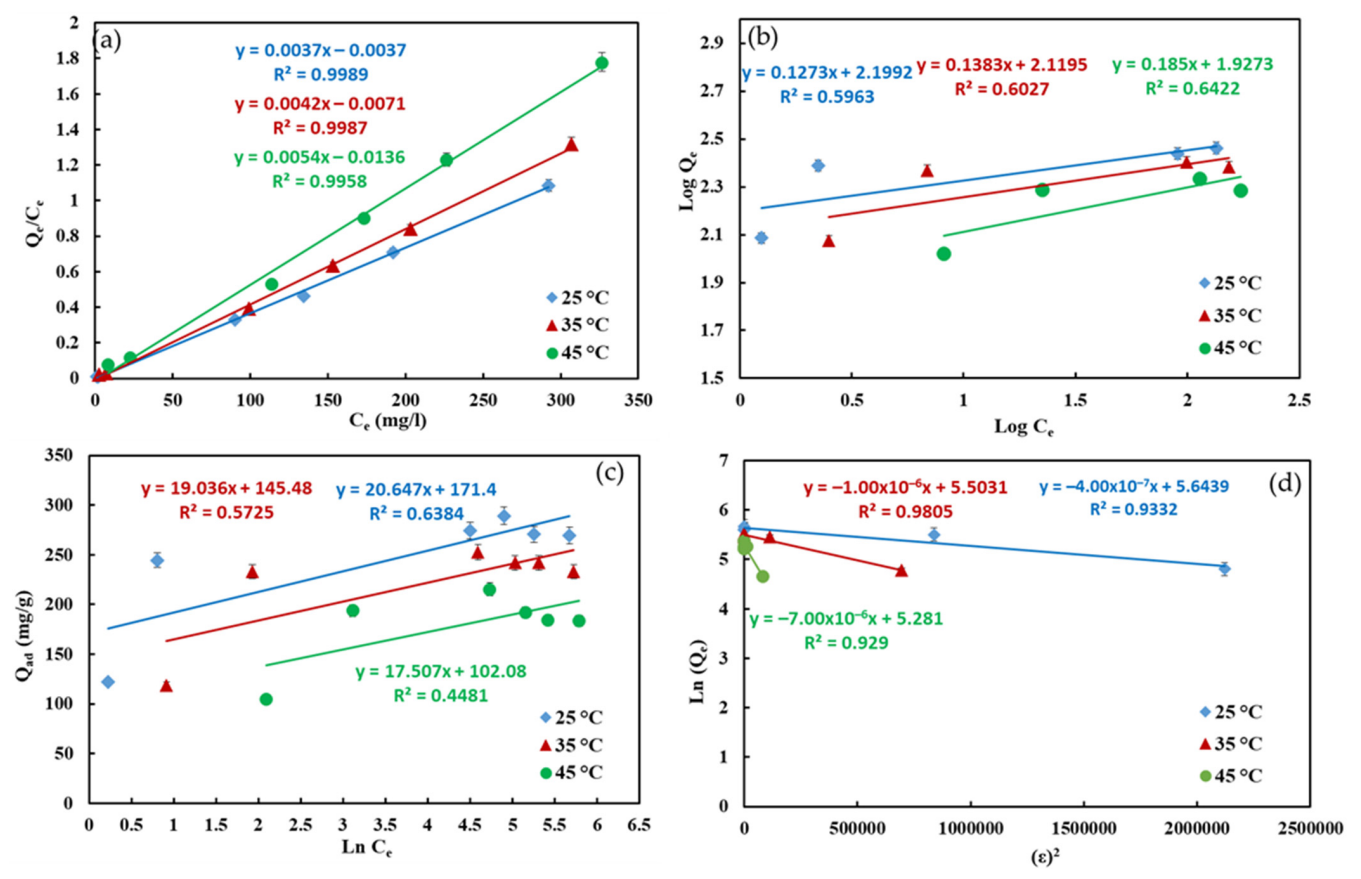
Linearized Irving Langmuir (**a**), Heurbet Freundlich (**b**), Mikhail Temkin (**c**), and Dubinin Radushkevich (**d**) isotherm adsorption models of CR dye onto CS-HAP-CeO_2_ heterostructure.

The linear plots of four isotherm models are shown in Figure 8, and the isotherm parameters are listed in Table 2. It was noticed that the R^2^ values of the Langmuir adsorption model were the highest (R^2^ = 0.9989) compared to the other isotherm models, Dubunin–Radushkevich (R^2^ = 0.9332), Temkin (R^2^ = 0.6384), and Freundlich (R^2^ = 0.5963). That means the adsorption process was well fitted with Langmuir adsorption model, representing monolayer adsorption on homogeneous surfaces. The maximum monolayer adsorption capacity of CS-HAP-CeO_2_ nanocomposite toward CR dye was 270.27 mg/g. This value is greater than the adsorption capacities of other adsorbents listed in Table 3 [34,56,57,58,59,60,61]. From the Dubinin–Radushkevich model, the magnitude of E is used for evaluating the type of adsorption mechanism. If the value of E is between 8 and 16 kJ/mol, it indicates chemisorption, while for values of E < 8 kJ/mol, the sorption process is of a physical nature [62,63]. Our results showed that the adsorption of CR on heterostructure adsorbent is physical adsorption, owing to the values of E being between 0.2672 and 1.118 kJ/mol (25 °C < T < 45 °C).

### 3.5. Thermodynamic Studies

**Figure 9:**
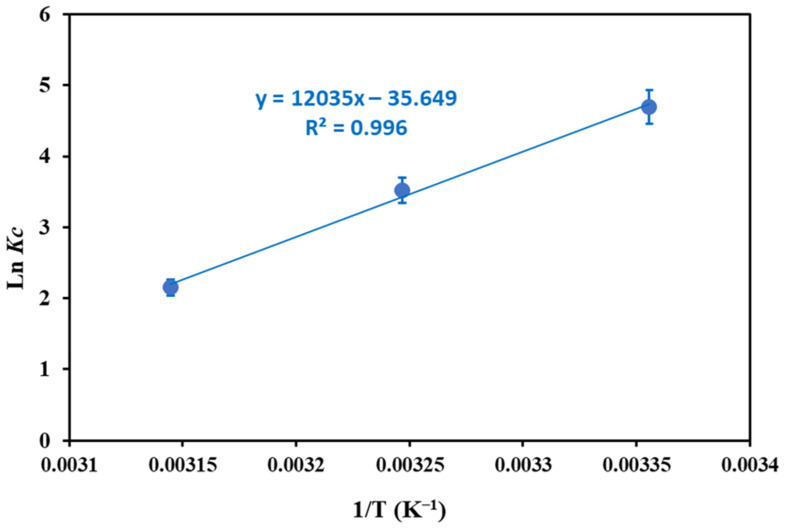
Plot of ln K vs. 1/T for thermodynamic parameters calculation.

To explain the effect of temperature on the CR adsorption over adsorbent and interpret whether the adsorption process occurs spontaneously or not, thermodynamic parameters including, free energy change (Δ*G*°), enthalpy change (Δ*H*°), and entropy change (Δ*S*°) were calculated using the following Equations (12) and (13) [64]:

(12) ΔG°=−RT lnKc

(13) $$\ln Kc=-\frac{\Delta H^{{}^{\circ}}}{RT}+\frac{\Delta S^{{}^{\circ}}}{R}$$

where k is *Kc* = *q_e_*/*C_e_*. The values of Δ*H*° and Δ*S*° were calculated from the slope and intercept of the plot ln kc versus 1/T (Figure 9). The thermodynamic parameters are summarized in Table 4. The negative values of Δ*G*° and Δ*H*° imply that the CR adsorption over CS-HAP-CeO_2_ heterostructure are spontaneous and exothermic [65]. The values of Δ*G*° slightly increased from −11.63 to −5.69 (kJ mol^−1^) with the rising temperature from 298 to 318 K, indicating the adsorption process is favored at lower temperatures [65]. The Δ*S*° negative value implies a decrease in the randomness of the solid-solution interface.

### 3.6. CR Dye Adsorption Mechanism

**Figure 10:**
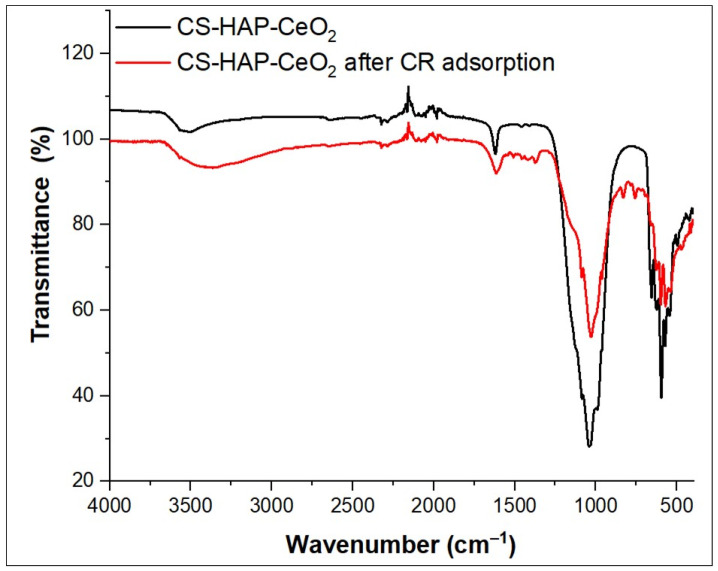
FTIR spectra of CS-HAP-CeO_2_ heterostructure before and after CR dye adsorption.

**Figure 11:**
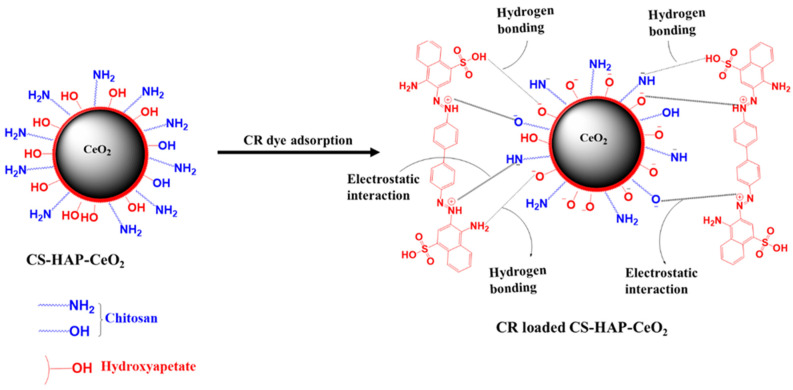
Mechanism of adsorption of CR onto CS-HAP-CeO_2_ heterostructure.

CS-HAP-CeO_2_ heterostructure adsorbent contains functional groups on their surfaces such as phosphate, amino, and hydroxyl groups which are responsible for the binding of the CR dye. According to the dissociation constant (pKa) of CR dye, when the pH < (pKa = 4), the surfaces of both CS-HAP-CeO_2_ heterostructure and CR dye are positively charged, representing an electrostatic repulsion between them. On the other hand, when the pH > (pKa = 4), the CR dissociates into polar groups (SO_3_^−^) along with the positive charge of the –N=N^+^– group. The SO_3_^−^ group over CR surface adsorbs on positively charged CS-HAP-CeO_2_ adsorbent by electrostatic attraction along with H-bonding. As pH increases to 7, the removal efficiency of CR increased due to the decrease in the number of positive charges in the CS-HAP-CeO_2_ heterostructure surface which led to the creation of electrostatic attraction between the –N=N^+^– group of CR molecules and the negative charge of CS-HAP-CeO_2_ heterostructure surface. The functional groups of CS-HAP-CeO_2_ heterostructure before/and after adsorbing CR dye were identified through FTIR analysis, and the obtained results are shown in Figure 10. It was observed that the intensity of O–H/NH_2_ stretching shifted from 3529 to 3382 cm^−1^ and the band associated with P-O at 1038 cm^−1^ decreased in intensity and shifted to 1029 cm^−1^ after CR dye adsorption. This is due to the interaction of –N=N^+^– and –NH_2_, –SO_3_H groups on the CR surface of dye with PO_4_^−3^ and O–H/NH_2_ groups on the surface of CS-HAP-CeO_2_ heterostructure through electrostatic attraction and H- bonding. The appearance of two new bands at 830 and 755 cm^−1^ after CR dye adsorption indicates the CR dye loaded successfully onto the CS-HAP-CeO_2_ heterostructure surface. Therefore, the mechanism of adsorption of CR dye onto CS-HAP-CeO_2_ heterostructure occurred by two mechanisms namely, electrostatic interaction and H-bonding (Figure 11). 

### 3.7. Antimicrobial 

**Figure 12:**
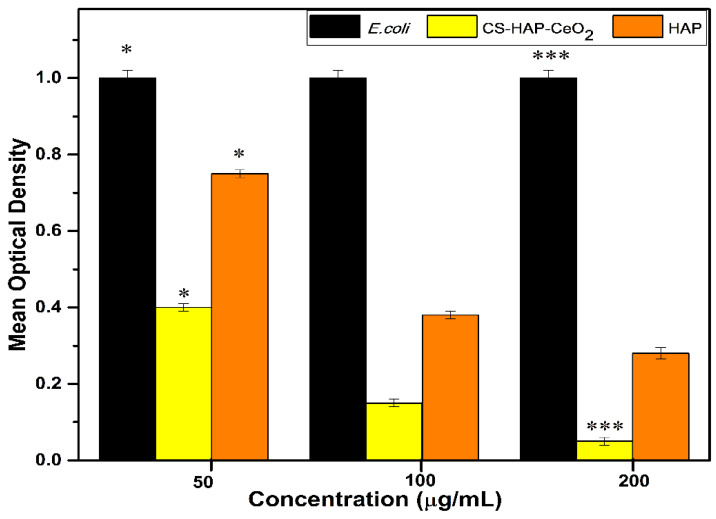
Bar illustrations demonstrating *E. coli* supplemented with different amounts of HAP and CS-HAP-CeO_2_ heterostructure (peak enlargement indicates *E. coli* deprived of HAP and CS-HAP-CeO_2_). Considerable difference (* *p* ≤ 0.05) was perceived between control and treatments. Substantial variance in bacteriostatic effect with HAP and CS-HAP-CeO_2_ at high quantity was envisaged. * *p* ≤ 0.05, *** *p* ≤ 0.001 suggestively different from virgin culture.

The virgin HAP and CS-HAP-CeO_2_ heterostructure were also evaluated using varying amounts against *E. coli* [66,67]. The produced heterostructure proved reputable antimicrobial activity against *E. coli* (MIC-50 μg/mL). The effects are shown in Figure 12. Alternatively, moderately trifling antibacterial achievement was detected with HAP at equal concentrations. Obviously, less bacteriostatic outcome was found at small amounts of HAP against *E. coli*, nonetheless improved activity was found with CS-HAP-CeO_2_ heterostructure. The greater activity of CS-HAP-CeO_2_ heterostructure was endorsed to morphological topographies with large surface [68] and synergism [69] between HAP and CeO_2_. The coating of CS also augmented in antimicrobial performance. The supposition about the antimicrobial action may be as: possibly at first HAP and CS-HAP-CeO_2_ heterostructure are interrelated with bacterial membrane, further disseminating into the inner cell prompting an outflow by disruption of the content of cell. Previous studies have described that nanocomposites via electrostatic attraction result in disruption of membrane, quash enzymes, break bacteria, ultimately hampering protein synthesis [70]. By and large, the bacteriostatic effect of HAP is correlated to liberation of OH^−^ ions in culture broth. Hydroxyl ions are tremendously oxidant free radicals which depict pronounced reactivity toward biomolecules. Furthermore, their lethal influence on bacterial cells are collectively allocated to aforesaid mechanisms [70]. 

## 4. Conclusions

In this paper, HAP and CS-HAP-CeO_2_ heterostructures were primed by hydrothermal method. The physicochemical data confirms efficacious formation of CS-HAP-CeO_2_ heterostructure. The adsorption results of CR dyes indicated outstanding efficiency of CS-HAP-CeO_2_ adsorbent. Additionally, the synthesized pristine and CS-HAP-CeO_2_ heterostructures unveiled good recyclable use and stability. The enhanced adsorption and antibacterial action of CS-HAP-CeO_2_ was mainly attributed to combined influence of CS, HAP and CeO_2_. Conclusively, outcomes of this study endorse an exceptionally capable adsorbent for decontamination of effluents; moreover, helps in reutilization of wastes of Baha city and meritoriously remove the environmental burden and govern the remediation procedure of the pathogenic diseases.

## Figures and Tables

**Table 1:** Kinetic parameters for the pseudo-first and pseudo-second-order adsorption model.

Model	Parameters	Value
	*C*_o_: 100 mg/L, *q_e,exp._:* 237.95 mg/g	
Pseudo-first-order	*q_e_*_1*, cal.*_ (mg/g)	98.01
	*k*_1_ (L/min)	0.360
	*R* ^2^	0.7967
Pseudo-second-order	*q_e_*_2*, cal.*_ (mg/g)	238.09
	*k*_2_ (g/mg-min)	0.0098
	*R* ^2^	0.9999

**Table 2:** Parameters for plotting Langmuir, Freundlich, Temkin, and Dubinin–Radushkevich adsorption isotherms of CR onto CS-HAP-CeO_2_ heterostructure.

Model	CR		
	298 K	308 K	318 K
*Langmuir*			
*q_m_*, mg/g	270.27	238.09	185.18
*K_L_* (L/mg)	1.000	0.591	0.397
*R* ^2^	0.9989	0.9987	0.9958
*Freundlich*			
*K_f_*,(mg/g) (L/mg)^1/*n*^	158.19	131.67	84.58
*N*	7.85	7.23	5.40
*R* ^2^	0.5963	0.6027	0.6422
*Dubinin-R*			
*q_s_*, mg/g	282.56	245.45	196.56
*K_D-R_* (mol^2^ KJ^−2^)	4.00 × 10^−7^	1.00 × 10^−6^	7.00 × 10^−6^
*E* (kJ mol^−1^)	1.118	0.7071	0.2672
*R* ^2^	0.9332	0.9805	0.929
*Temkin*			
*b_T_ = RT/B*	119.99	130.15	141.519
*A_T_* (L/g)	4029.70	2084.66	340.63
*B*	20.647	19.036	17.507
*R* ^2^	0.6384	0.5725	0.4481

**Table 3:** Comparison of the maximum adsorption capacities for CR adsorption with different adsorbents.

Adsorbent	*q_m_* (mg/g)	Isotherm/Kinetic Models	Ref.
CeO_2_ nanocrystals	237	Langmuir/PSO	[56]
AgNPs-functionalized HAP	159.11	Freundlich/PSO	[57]
CS@GO-Hap composite	43.06	Freundlich/PSO	[58]
Hydroxyapatite (HAp)	139 mg/g	Freundlich/PSO	[59]
ZnO/chitosan	227.3	Langmuir	[60]
Ground nut shells charcoal (GNC)	117.6	Freundlich/PSO	[34]
Eichhornia charcoal (EC)	56.8	Freundlich/PSO	[34]
Polygorskite -700T	136.1	Freundlich/Elovich	[61]
CS-HAP-CeO_2_	270.27	Langmuir/PSO	This study

**Table 4:** Thermodynamic parameters for the adsorption of CV on CS-HAP-CeO_2_ heterostructure adsorbent.

*C_o_* (mg/L)	(−) ΔH° (kJ/mol)	(−) ΔS° (J/mol.K)	(−) ΔG° (kJ/mol)		
			298 K	308 K	318 K
100	19.43	8.29	11.63	9.02	5.69

## Data Availability

The data is included in the main text.

## References

[1] Helaly M.N., El-Metwally M.A., El-Hoseiny H., Omar S.A., El-Sheery N.I. (2014). Effect of nanoparticles on biological contamination of ‘in vitro’cultures and organogenic regeneration of banana. Aust. J. Crop Sci..

[2] Nawab J., Khan S., Khan M.A., Sher H., Rehamn U.U., Ali S., Shah S.M. (2017). Potentially toxic metals and biological contamination in drinking water sources in chromite mining-impacted areas of Pakistan: A comparative study. Expo. Health.

[3] Pathania D., Sharma A., Siddiqi Z.-M. (2016). Removal of congo red dye from aqueous system using Phoenix dactylifera seeds. J. Mol. Liq..

[4] Sathishkumar K., Alsalhi M.S., Sanganyado E., Devanesan S., Arulprakash A., Rajasekar A. (2019). Sequential electrochemical oxidation and bio-treatment of the azo dye congo red and textile effluent. J. Photochem. Photobiol. B Biol..

[5] Dawlet A., Talip D., Mi H.Y. (2013). Removal of mercury from aqueous solution using sheep bone charcoal. Procedia Environ. Sci..

[6] Alqadami A.A., Khan M.A., Otero M., Siddiqui M.R., Jeon B.-H., Batoo K.M. (2018). A magnetic nanocomposite produced from camel bones for an efficient adsorption of toxic metals from water. J. Clean. Prod..

[7] Panneerselvam K., Arul K.T., Warrier A.R., Asokan K., Dong C.-L. (2019). Rapid adsorption of industrial pollutants using metal ion doped hydroxyapatite. AIP Conf. Proc..

[8] Manatunga D.C., De Silva R.M., De Silva K.N., De Silva N., Premalal E. (2018). Metal and polymer-mediated synthesis of porous crystalline hydroxyapatite nanocomposites for environmental remediation. R. Soc. Open Sci..

[9] Guan Y., Cao W., Guan H., Lei X., Wang X., Tu Y., Marchetti A., Kong X. (2018). A novel polyalcohol-coated hydroxyapatite for the fast adsorption of organic dyes. Colloids Surf. A Physicochem. Eng. Asp..

[10] Fihri A., Len C., Varma R.S., Solhy A. (2017). Hydroxyapatite: A review of syntheses, structure and applications in heterogeneous catalysis. Coord. Chem. Rev..

[11] Piccirillo C., Castro P. (2017). Calcium hydroxyapatite-based photocatalysts for environment remediation: Characteristics, performances and future perspectives. J. Environ. Manag..

[12] Tommalieh M., Ibrahium H.A., Awwad N.S., Menazea A. (2020). Gold nanoparticles doped polyvinyl alcohol/chitosan blend via laser ablation for electrical conductivity enhancement. J. Mol. Struct..

[13] Bagheri A.R., Ghaedi M. (2020). Green preparation of dual-template chitosan-based magnetic water-compatible molecularly imprinted biopolymer. Carbohydr. Polym..

[14] Al-Kahtani S.H., Sofian B.E.-D.E. (1995). Estimating preference change in meat demand in Saudi Arabia. Agric. Econ..

[15] Gholami H., Ghaedi M., Arabi M., Ostovan A., Bagheri A.R., Mohamedian H. (2019). Application of molecularly imprinted biomembrane for advancement of matrix solid-phase dispersion for clean enrichment of parabens from powder sunscreen samples: Optimization of chromatographic conditions and green approach. ACS Omega.

[16] Arabi M., Ostovan A., Bagheri A.R., Guo X., Wang L., Li J., Wang X., Li B., Chen L. (2020). Strategies of molecular imprinting-based solid-phase extraction prior to chromatographic analysis. TrAC Trends Anal. Chem..

[17] Menazea A., Eid M., Ahmed M. (2020). Synthesis, characterization, and evaluation of antimicrobial activity of novel Chitosan/Tigecycline composite. Int. J. Biol. Macromol..

[18] Madni A., Kousar R., Naeem N., Wahid F. (2021). Recent advancements in applications of chitosan-based biomaterials for skin tissue engineering. J. Bioresour. Bioprod..

[19] Saad E.M., Elshaarawy R.F., Mahmoud S.A., El-Moselhy K.M. (2021). New ulva lactuca algae based chitosan bio-composites for bioremediation of Cd (II) ions. J. Bioresour. Bioprod..

[20] Jian S., Tian Z., Zhang K., Duan G., Yang W., Jiang S. (2021). Hydrothermal synthesis of Ce-doped ZnO heterojunction supported on carbon nanofibers with high visible light photocatalytic activity. Chem. Res. Chin. Univ..

[21] Al-Shwaiman H.A., Akshhayya C., Syed A., Bahkali A.H., Elgorban A.M., Das A., Varma R.S., Khan S.S. (2022). Fabrication of intimately coupled CeO_2_/ZnFe_2_O_4_ nano-heterojunction for visible-light photocatalysis and bactericidal application. Mater. Chem. Phys..

[22] Tomic N.M., Dohcevic-Mitrovic Z.D., Paunović N.M., Mijin D.A.Z., Radić N.D., Grbic B.V., Askrabic S.M., Babić B.M., Bajuk-Bogdanovic D.V. (2014). Nanocrystalline CeO_2−δ_ as effective adsorbent of azo dyes. Langmuir.

[23] Jian S., Shi F., Hu R., Liu Y., Chen Y., Jiang W., Yuan X., Hu J., Zhang K., Jiang S. (2022). Electrospun magnetic La_2_O_3_–CeO_2_–Fe_3_O_4_ composite nanofibers for removal of fluoride from aqueous solution. Compos. Commun..

[24] Amna T. (2018). Valorization of bone waste of Saudi Arabia by synthesizing hydroxyapatite. Appl. Biochem. Biotechnol..

[25] Selim S.E., Meligi G.A., Abdelhamid A.E., Mabrouk M.A., Hussain A.I. (2022). Novel Composite Films Based on Acrylic Fibers Waste/Nano-chitosan for Congo Red Adsorption. J. Polym. Environ..

[26] Amna T., Hassan M.S., Barakat N.A., Pandeya D.R., Hong S.T., Khil M.-S., Kim H.Y. (2012). Antibacterial activity and interaction mechanism of electrospun zinc-doped titania nanofibers. Appl. Microbiol. Biotechnol..

[27] Guvensen N.C., Demir S., Ozdemir G. (2012). Effects of magnesium and calcium cations on biofilm formation by Sphingomonas Paucimobilis from an industrial environment. Fresenius Environ. Bull..

[28] Djuričić B., Pickering S. (1999). Nanostructured cerium oxide: Preparation and properties of weakly-agglomerated powders. J. Eur. Ceram. Soc..

[29] El Boujaady H., Mourabet M., El Rhilassi A., Bennani-Ziatni M., El Hamri R., Taitai A. (2016). Adsorption of a textile dye on synthesized calcium deficient hydroxyapatite (CDHAp): Kinetic and thermodynamic studies. J. Mater. Environ. Sci..

[30] Nusrath K., Muraleedharan K. (2016). Synthesis, characterization and thermal decomposition kinetics of cerium oxalate rods. Devagiri J. Sci..

[31] Kumar S., Koh J. (2012). Physiochemical, optical and biological activity of chitosan-chromone derivative for biomedical applications. Int. J. Mol. Sci..

[32] Anirudhan T., Ramachandran M. (2015). Adsorptive removal of basic dyes from aqueous solutions by surfactant modified bentonite clay (organoclay): Kinetic and competitive adsorption isotherm. Process Saf. Environ. Prot..

[33] Allen S.J., Mckay G., Khader K. (1989). Equilibrium adsorption isotherms for basic dyes onto lignite. J. Chem. Technol. Biotechnol..

[34] Kaur S., Rani S., Mahajan R.K. (2013). Adsorption Kinetics for the Removal of Hazardous Dye Congo Red by Biowaste Materials as Adsorbents. J. Chem..

[35] Purkait M.K., Maiti A., Dasgupta S., De S. (2007). Removal of congo red using activated carbon and its regeneration. J. Hazard. Mater..

[36] Phạm V.T., Hong-Tham N., Tran T., Nguyen D., Le H., Nguyen T., Vo D.-V., Le N., Nguyen D.C. (2019). Kinetics, Isotherm, Thermodynamics, and Recyclability of Exfoliated Graphene-Decorated MnFe_2_O_4_ Nanocomposite Towards Congo Red Dye. J. Chem..

[37] Astuti D., Aprilita N., Mudasir M. (2020). Adsorption of the anionic dye of congo red from aqueous solution using a modified natural zeolite with benzalkonium chloride. Rasayan J. Chem..

[38] Parvin S., Hussain M., Akter F., Biswas B. (2021). Removal of Congo Red by Silver Carp (Hypophthalmichthys molitrix) Fish Bone Powder: Kinetics, Equilibrium, and Thermodynamic Study. J. Chem..

[39] Jiang H., Cao Y., Zeng F., Xie Z., He F. (2021). A Novel Fe_3_O_4_/Graphene Oxide Composite Prepared by Click Chemistry for High-Efficiency Removal of Congo Red from Water. J. Nanomater..

[40] Alam G., Ihsanullah I., Naushad M., Sillanpää M. (2022). Applications of artificial intelligence in water treatment for optimization and automation of adsorption processes: Recent advances and prospects. Chem. Eng. J..

[41] Chen Y., Hanshe M., Sun Z., Zhou Y., Mei C., Duan G., Zheng J., Shiju E., Jiang S. (2022). Lightweight and anisotropic cellulose nanofibril/rectorite composite sponges for efficient dye adsorption and selective separation. Int. J. Biol. Macromol..

[42] Çiner F. (2018). Application of Fenton reagent and adsorption as advanced treatment processes for removal of Maxilon Red GRL. Glob. Nest J..

[43] Shoukat S., Bhatti H.N., Iqbal M., Noreen S. (2017). Mango stone biocomposite preparation and application for crystal violet adsorption: A mechanistic study. Microporous Mesoporous Mater..

[44] Alamrani N., Al_Aoh H. (2021). Elimination of Congo Red Dye from Industrial Wastewater Using Teucrium polium L. as a Low-Cost Local Adsorbent. Adsorpt. Sci. Technol..

[45] Zhang F., Yin Y., Qiao C., Luan Y.-N., Guo M., Xiao Y., Liu C. (2021). Anionic Dye Removal by Polypyrrole-Modified Red Mud and Its Application to a Lab-Scale Column: Adsorption Performance and Phytotoxicity Assessment. Adsorpt. Sci. Technol..

[46] Omidi S., Kakanejadifard A. (2018). Eco-friendly synthesis of graphene–chitosan composite hydrogel as efficient adsorbent for Congo red. RSC Adv..

[47] Pirbazari A.E., Saberikhah E., Badrouh M., Emami M.S. (2014). Alkali treated Foumanat tea waste as an efficient adsorbent for methylene blue adsorption from aqueous solution. Water Resour. Ind..

[48] Nasuha N., Hameed B., Din A.T.M. (2010). Rejected tea as a potential low-cost adsorbent for the removal of methylene blue. J. Hazard. Mater..

[49] Al-Salihi S., Jasim A.M., Fidalgo M.M., Xing Y. (2022). Removal of Congo red dyes from aqueous solutions by porous γ-alumina nanoshells. Chemosphere.

[50] Al-Shehri H.S., Almudaifer E., Alorabi A.Q., Alanazi H.S., Alkorbi A.S., Alharthi F.A. (2021). Effective adsorption of crystal violet from aqueous solutions with effective adsorbent: Equilibrium, mechanism studies and modeling analysis. Environ. Pollut. Bioavailab..

[51] Ding L., Li B., Mi J. (2014). The Effective Removal of Congo Red Dye from Aqueous Solution Using Fly Ash/CeO_2_ Composite Material. Appl. Mech. Mater..

[52] Zhang F., Ma B., Jiang X., Ji Y. (2016). Dual function magnetic hydroxyapatite nanopowder for removal of malachite green and Congo red from aqueous solution. Powder Technol..

[53] Du Q., Sun J., Li Y., Yang X., Wang X., Wang Z., Xia L. (2014). Highly enhanced adsorption of congo red onto graphene oxide/chitosan fibers by wet-chemical etching off silica nanoparticles. Chem. Eng. J..

[54] Chahkandi M. (2017). Mechanism of Congo red adsorption on new sol-gel-derived hydroxyapatite nano-particle. Mater. Chem. Phys..

[55] Alqadami A.A., Naushad M., Abdalla M.A., Khan M.R., Alothman Z.A. (2016). Adsorptive removal of toxic dye using Fe_3_O_4_–TSC nanocomposite: Equilibrium, kinetic, and thermodynamic studies. J. Chem. Eng. Data.

[56] Rao R., Jin P., Huang Y., Hu C., Dong X., Tang Y., Wang F., Luo F., Fang S. (2022). A surface control strategy of CeO_2_ nanocrystals for enhancing adsorption removal of Congo red. Colloid Interface Sci. Commun..

[57] Azeez L., Adebisi S.A., Adejumo A.L., Busari H.K., Aremu H.K., Olabode O.A., Awolola O. (2022). Adsorptive properties of rod-shaped silver nanoparticles-functionalized biogenic hydroxyapatite for remediating methylene blue and congo red. Inorg. Chem. Commun..

[58] Sirajudheen P., Karthikeyan P., Ramkumar K., Meenakshi S. (2020). Effective removal of organic pollutants by adsorption onto chitosan supported graphene oxide-hydroxyapatite composite: A novel reusable adsorbent. J. Mol. Liq..

[59] Bensalah H., Younssi S.A., Ouammou M., Gurlo A., Bekheet M.F. (2020). Azo dye adsorption on an industrial waste-transformed hydroxyapatite adsorbent: Kinetics, isotherms, mechanism and regeneration studies. J. Environ. Chem. Eng..

[60] Nguyen N.T., Nguyen N.T., Nguyen V.A. (2020). In Situ Synthesis and Characterization of ZnO/Chitosan Nanocomposite as an Adsorbent for Removal of Congo Red from Aqueous Solution. Adv. Polym. Technol..

[61] Silva V.C., Araújo M.E.B., Rodrigues A.M., Vitorino M.D.B.C., Cartaxo J.M., Menezes R.R., Neves G.A. (2021). Adsorption Behavior of Crystal Violet and Congo Red Dyes on Heat-Treated Brazilian Palygorskite: Kinetic, Isothermal and Thermodynamic Studies. Materials.

[62] El Haddad M. (2016). Removal of Basic Fuchsin dye from water using mussel shell biomass waste as an adsorbent: Equilibrium, kinetics, and thermodynamics. J. Taibah Univ. Sci..

[63] Mladenovic N., Petkovska J., Dimova V., Dimitrovski D., Jordanov I. (2022). Circular economy approach for rice husk modification: Equilibrium, kinetic, thermodynamic aspects and mechanism of Congo red adsorption. Cellulose.

[64] Cui M., Li Y., Sun Y., Wang H., Li M., Li L., Xu W. (2021). Study on adsorption performance of MgO/Calcium alginate composite for congo red in wastewater. J. Polym. Environ..

[65] Extross A., Waknis A., Tagad C., Gedam V., Pathak P. (2022). Adsorption of congo red using carbon from leaves and stem of water hyacinth: Equilibrium, kinetics, thermodynamic studies. Int. J. Environ. Sci. Technol..

[66] Amna T., Hassan M.S., El-Newehy M.H., Alghamdi T., Moydeen Abdulhameed M., Khil M.-S. (2021). Biocompatibility Computation of Muscle Cells on Polyhedral Oligomeric Silsesquioxane-Grafted Polyurethane Nanomatrix. Nanomaterials.

[67] Amna T., Alghamdi A.A., Shang K., Hassan M.S. (2021). Nigella Sativa-Coated Hydroxyapatite Scaffolds: Synergetic Cues to Stimulate Myoblasts Differentiation and Offset Infections. Tissue Eng. Regen. Med..

[68] Ann L.C., Mahmud S., Bakhori S.K.M., Sirelkhatim A., Mohamad D., Hasan H., Seeni A., Rahman R.A. (2014). Antibacterial responses of zinc oxide structures against Staphylococcus aureus, Pseudomonas aeruginosa and Streptococcus pyogenes. Ceram. Int..

[69] Soletti L.D.S., Ferreira M.E.C., Kassada A.T., Abreu Filho B.a.D., Bergamasco R., Yamaguchi N.U. (2020). Manganese ferrite graphene nanocomposite synthesis and the investigation of its antibacterial properties for water treatment purposes. Rev. Ambiente Água.

[70] Goudarzi M.R., Bagherzadeh M., Fazilati M., Riahi F., Salavati H., Esfahani S.S. (2019). Evaluation of antibacterial property of hydroxyapatite and zirconium oxide-modificated magnetic nanoparticles against Staphylococcus aureus and *Escherichia coli*. IET Nanobiotechnol..

